# QD:Puf Nanohybrids Are Compatible with Studies in Cells

**DOI:** 10.3390/nano12183174

**Published:** 2022-09-13

**Authors:** Karolina Wójtowicz, Magda A. Antoniak, Martyna Trojnar, Marcin Nyk, Tomasz Trombik, Joanna Grzyb

**Affiliations:** 1 Department of Biotransformation, Faculty of Biotechnology, University of Wrocław, ul. F. Joliot-Curie 14a, 50-383 Wrocław, Poland; 2 Advanced Materials Engineering and Modelling Group, Faculty of Chemistry, Wrocław University of Science and Technology, Wybrzeże Wyspiańskiego 27, 50-370 Wrocław, Poland; 3 Department of Biophysics, Faculty of Biotechnology, University of Wrocław, ul. F. Joliot-Curie 14a, 50-383 Wrocław, Poland; 4 The Chair and Department of Biochemistry and Molecular Biology, Medical University of Lublin, ul. Chodzki 1, 20-093 Lublin, Poland

**Keywords:** quantum dots, Puf protein, nanohybrids, HeLa cells

## Abstract

Colloidal semiconductor quantum dots (QD), as well as other nanoparticles, are useful in cell studies as fluorescent labels. They may also be used as more active components in various cellular assays, serving as sensors or effectors. However, not all QDs are biocompatible. One of the main problems is their outer coat, which needs to be stable and to sustain hydrophilicity. Here we show that purpose-designed CdSe QDs, covered with a Puf protein, can be efficiently accumulated by HeLa cells. The uptake was measurable after a few hours of incubation with nanoparticles and most of the fluorescence was localised in the internal membrane system of the cell, including the endoplasmic reticulum and the Golgi apparatus. The fluorescence properties of QDs were mostly preserved, although the maximum emission wavelength was slightly shifted, and the fluorescence lifetime was shortened, indicating partial sensitivity of the QDs to the cell microenvironment. QD accumulation resulted in a decrease in cell viability, which was attributed to disturbance of endoplasmic reticulum performance.

## 1. Introduction

Quantum dots (QD) are colloidal, semiconductor nanocrystals, widely known due to their bright fluorescence. Most QDs are approximately nanospheres (1 to 10 nm diameter). The size of the sphere imposes spatial confinement effects and affects the extent of the energy gap. Consequently, the diameter of QDs defines their emission maxima. The most useful are QDs which emit in the visible light range (e.g., CdTe, CdSe), although there are some QDs able to emit in the infrared window (e.g., PbS, Pb:Ag2S) [1,2]. There are other types of nanostructures of similar size, composed of different materials; some are fluorescent, while others offer plasmonic enhancement of absorption or fluorescence quenching only. Here, we used fluorescent CdSe QDs emitting in a green part of the light spectrum, with a maximum at about 560 nm. 

The spectroscopic properties of QDs, namely their narrow emission band, broad absorption generating huge Stokes shifts, and resistance to photobleaching, make them perfect tools for labeling and tracking in cell studies. On the other hand, QDs built of cadmium and other heavy metals show high cytotoxicity. This is unsurprising given the possibility of nanocrystal decomposition and the release of toxic ions. A further problem is the actual delivery of QDs to cells. Many QD synthesis routes result in the formation of hydrophobic nanocrystals, which are non-mixable with the cellular medium and, therefore, are not accessible to cells. Hydrophobic QDs must be converted into water-dispersible QDs if intended for cellular studies. There are several strategies for such conversion, starting from the simple substitution of a hydrophobic coat by small hydrophilic molecules, such as thiocarboxylates or, less often, thioamines. QDs may also be covered with long amphiphilic polymers [3] or embedded in silica [4]. We have developed a method for covering hydrophobic CdSe QDs with proteins [5,6]. However, there is still no perfect solution for covering QDs. The coat needs to enable QD uptake by cells, as well as provide protection from decomposition and the release of toxic ions. The cover may determine the fate of the nanoparticle in the cell, so it must be carefully selected for particular applications. If the resulting QD is too large, it will stick on the plasma membrane. If the cover is not stable enough, QDs may decompose and aggregate. There are several strategies for QD coat preparation. Attachment of protein as the outer shell may improve the potential for application of nanoparticles [7,8,9]. Protein-based nanoparticles may work as nanozymes and biosensors [10,11,12]. 

The CdSe QDs used in the present study are produced as hydrophobic nanocrystals covered with trioctylphosphine oxide (TOPO). To convert them into water-dispersible particles, we used derivatives of apolipoprotein A [6] or a Puf protein, based on the Pumilio homology domain motif [5]. The Puf motif is mostly found in DNA binding proteins. The Puf protein is composed of multiple α-helices, forming a broad ribbon embracing the QD nanocrystal sphere. The α-helical ribbon was found to be better in such applications than the β-sheet ribbon [5]. As a result, a QD:Puf nanohybrid of approximate radius 7 nm (determined by fluorescence) was produced. We have shown that a QD:Puf nanohybrid preserved the fluorescent properties of the original raw QDs, including upconversion/two-photon excitation [13]. Here, we assess whether QDs in a Puf cover are compatible with studies on living cells, namely that QD:Puf nanohybrids are (i) highly dispersible in the cellular medium with good colloidal stability, (ii) absorbed by cells, and (iii) stable inside the cells, with preserved optical properties. Additionally, we demonstrate that cytotoxicity is minimized when the small size of the delivered nanohybrid is assured.

## 2. Materials and Methods

### 2.1. Quantum Dots and Proteins

Quantum dots (2.8 nm core diameter) were prepared by a thermal decomposition method, as described in [13]. The morphology (see Supplementary Materials, Figure S1) of the raw QDs was studied by transmission electron microscopy (TEM) on a Tecnai G^2^ 20 X-TWIN microscope (Fei, Hilsboro, OR, USA). As-prepared QDs were dispersed in chloroform, with an emission maximum of 560 nm. Puf protein was expressed in *E.coli* Bl21 and purified from inclusion bodies, as described in [5]. QD:Puf nanohybrid particles were obtained as previously described [5]. Briefly, SDS solubilized QDs were mixed with Puf proteins (1:20 QD:protein ratio), followed by dialysis against 25 mM Tris/HCl, pH 8.0 with 100 mM NaCl. The dialyzed mixture was then separated on a Superdex 200 10/100 column (GE Healthcare, Chicago, IL, USA) and equilibrated with the same buffer. Fractions were collected based on absorption at 280 nm and 550 nm. Excess protein, not assembled with QD, was eluted at higher elution times (at about 17.5 mL, see Figure S2)

QD, Puf and QD:Puf concentrations were determined on the basis of absorption spectra, with extinction coefficients of ε_553_ = 117,431 M^−1^cm^−1^ (for QD) and ε_280_ = 33,640 M^−1^cm^−1^ for Puf, respectively [5]. The QD:Puf concentration was estimated based on QD absorption. 

### 2.2. Cell Culture and QD:Puf Treatment 

HeLa and A375 cells were cultured in Dulbecco’s Modified Eagle’s Medium (DMEM) supplemented with 10% fetal bovine serum, penicillin (100 u/mL), streptomycin (100 u/mL) and L-glutamine (1 mM). The cells were cultured in a humidified atmosphere at 37 °C, with 5% CO_2_.

To determine the influence of QD:Puf on HeLa cells, the culture medium was removed and replaced by fresh complete DMEM containing an appropriate QDs dilution. The cells were then incubated for 24 h at 37 °C, in 5% CO_2_ and subjected to further analysis. 

### 2.3. Endocytosis Inhibition

For endocytosis inhibition tests, the temperature of incubation with QD:Puf was lowered to 22 °C, while the other parameters of cell incubation were kept unchanged. The CLSM images collected for this experiment were analyzed with Fiji software [14]. At least 20 cells for both conditions were analyzed using the plugin “Particle analysis”. Total cell accumulated QD:Puf fluorescence, as well as the area (the pixel count) of this fluorescence, were calculated to provide a cell-size-independent comparison of the ratio of these two values. 

### 2.4. Toxicity Tests

The toxicity of Puf-QDs towards HeLa cells was assessed by MTT assay. For this purpose, the cells were seeded in triplicate in 96-well plates at 5 × 10^3^ cells/well or 1 × 10^3^ cells/well and cultured overnight in a complete DMEM medium at 37 °C, with 5% CO_2_. The medium was then removed and replaced by a QD:Puf preparation diluted in fresh culture medium (10–0.3125 nM). The cells were incubated for 24 h at 37 °C, with 5% CO_2_. After the incubation, the mixture was discarded, and the cells were washed before the addition of 100 µL of MTT reagent (0.5 mg/mL, Sigma-Aldrich, Darmstadt, Germany) in a complete DMEM medium to each well. The cells were then incubated for 3 h at 37 °C, with 5% CO_2_. Finally, the solution was carefully removed and the formazan crystals were dissolved by adding 100 µL of DMSO. The plates were shaken for 10 min and the absorbance was measured using a UVM 340 microplate reader (Biogenet, Józefów, Poland) at 550 nm with a reference wavelength of 630 nm. The values obtained for the untreated controls were normalized to 100% and the viability of treated cells was estimated as the percentage of the control. 

### 2.5. Spectrophotometry and Spectrofluorometry

Absorption spectra were determined with a DU 800 spectrophotometer (Beckman, Brea, CA, USA). Steady-state emission and time-resolved fluorescence decays were recorded with an FS5 spectrofluorometer (Edinburg Instruments, Livingston, UK), using excitation with a xenon continuous lamp or a pulse laser (471 nm), respectively. Data were processed with Fluoracle software (Edinburg Instruments, Livingston, UK).

### 2.6. Confocal Laser Scanning Microscopy (CLSM)

CLSM and FLIM images were obtained using the Stellaris (Leica) platform. For most cases, a white pulse laser was used, and the precise excitation wavelength was given in the image description. For Laurdan-stained cells, a 405 nm diode laser was used. All cell images were obtained directly in growing chambers of borosilicate glass (LabTek system). The collected data were processed and analyzed with LasX and FLIM LasX software (Leica, Wetzlar, Germany).

Colocalization (Spearman and Manders’ coefficients) was determined using the Fiji software Coloc2 plugin.

### 2.7. Cell Staining

For microscopy imaging, HeLa cells were grown in 8-well LabTek chambers (Nunc) and seeded at 2 × 10^4^ cells/well. The cells were cultured overnight and fluorescently stained immediately before the observations. For this purpose, the cells were washed with PBS buffer and fixed with 4% formaldehyde for 20 min. at room temperature. Then the cells were washed with PBS buffer and stained simultaneously with 2.5 µg/mL of propidium iodide and 10 µg/mL of Laurdan in PBS buffer (5 min, room temperature). Finally, the cells were rinsed several times and maintained in PBS buffer while imaging was performed.

For immunofluorescence, the formaldehyde-fixed cells were permeablized for 15 min with 0.05% Triton in PBS, followed by washing with PBS (three times). Then cells were blocked by 1%BSA in PBS (1 h) and incubated for 3 h with primary antibodies diluted (1:100) in 1% BSA in PBS. Here, we used anti-calnexin (catalogue number MA3-027, Invitrogen) for staining ER and anti-golgin 97 (A-21270, Thermo Fisher, Waltham, MA, USA) for visualization of the Golgi apparatus. After washing (3 times with PBST, PBS + 0.05% Tween 20), cells were incubated (1 h) with secondary antibodies (goat-anti-mouse IgG labelled with Alexa647, A32728, Thermo Fischer) diluted (1:2000) in PBS with 1% BSA. Finally, cells were washed again three times with PBST and once (5 min) with PBS containing DAPI (2-(4-Amidinophenyl)-6-indolecarbamidine 5 µg/mL). The last was added to visualize the nucleus, as propidium iodide emission overlaps with Alexa Fluor 647.

### 2.8. IncuCyte Imaging

The penetration of QD:Puf into HeLa cells was monitored using the IncuCyte^®^ ZOOM system (Essen BioScience, Ann Arbor, MI, United States). HeLa cells were seeded in 24-well plates at 5 × 10^4^ cells/well and cultured overnight to allow the cells to attach to the plate. The next day, the culture medium was replaced by a complete DMEM medium (no phenol red) containing the appropriate concentration of QD:Puf. The cells were incubated for 24 h at 37 °C, and 5% CO_2_ and image sets were collected every 2 h. The obtained data were analyzed using Fiji software [14]. The analyzed spots were inspected and if necessary, corrected, for cell movement and the presence of artefacts of a detection system.

IncuCyte images were also used for the analysis of cell number changes upon QD:Puf treatment. For this, images series regions with 30–50 cells and relatively low starting confluence (20–30%) were selected. Cells were counted using the Fiji software CellCounter plugin.

### 2.9. Statistical Analysis

Statistical analysis was performed using one-way ANOVA with Tukey test.

## 3. Results and Discussion

### 3.1. Optimization of QD:Puf Concentration for In-Cell Delivery

**Figure 1:**
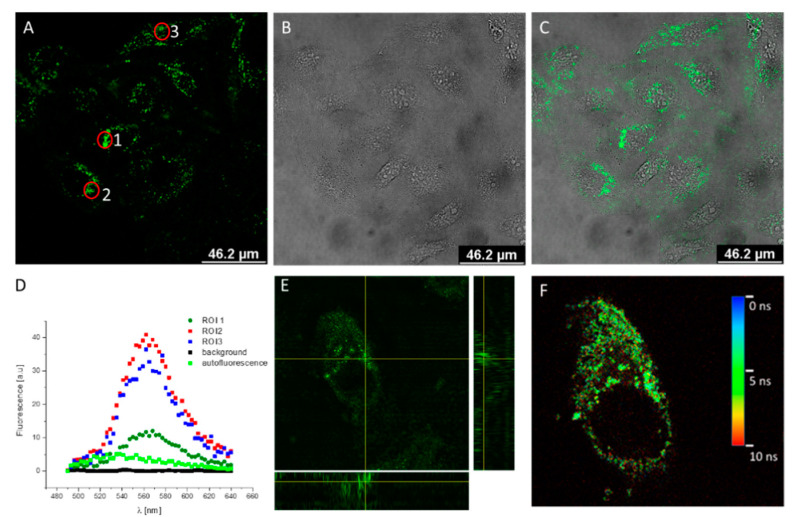
QD:Puf nanohybrids visualized inside HeLa cells after 24 h of incubation. CLSM images (**A**) fluorescence emission (excitation 450 nm, emission 550–650 nm), (**B**) bright-field and (**C**) image overlay. (**D**) emission spectra recorded for ROIs marked as the red circle in the image (**A**), background and control emission detected in untreated, control cells. (**E**) orthoslices of Z-stack recorded for individual treated cells. Z-stack were deconvolved and particular original Z-stack images are provided in Supplementary Materials, Figure S5. (**F**) FLIM image recorded for individual treated cells using 450 nm laser line and 550–650 nm emission range. The color scale represents individual pixel lifetime. The growth medium contained 10 nM of QD:Puf.

To check whether QD:Puf may have entered the HeLa cells, nanohybrid preparation was added to the cell medium directly and incubated under the previous growth conditions. We tested several dilutions of the QD:Puf preparation (1–20 nM). Additionally, we investigated whether the size of the nanohybrid mattered for in-cell delivery. For this purpose, we chose a fraction, corresponding to a single QD embraced by Puf (later termed the “M” fraction for “monomeric QD”, ~14 nm total diameter), and a fraction of larger particles (referred to as “O”, for “oligomers”, 20–30 nm diameter). Both O and M preparations were stable in the cell medium. This was demonstrated by fluorescence emission monitoring of QD:Puf mixed with the cell medium. No intensity changes were noted during the first 6 h incubation at 37 °C and only a 10–15% reduction in fluorescence intensity was noted after 24 h of incubation (Supplementary Materials, Figure S3). For the origin of O and M, see Supplementary Materials, Figure S2. As QDs are fluorescent, they may be simply visualized inside a cell with confocal microscopy (CLSM), using the properly selected excitation wavelength and emission range. Here, a preparation of QD:Puf, measured before administration to cells, had a characteristic emission maximum at 560 nm (Figure S4A). We found that, after 24 h incubation, QD fluorescence was detectable in cells for 5–20 nM QD:Puf in the growth medium. For further studies, we employed 10 nM QD:Puf (Figure 1), as it gave a clear fluorescence signal and acceptable toxicity (see further points). The range of concentrations was similar to that administrated in other studies [15,16].

Localization of QD:Puf inside a cell was confirmed by detailed Z-stack projection analysis (Figure 1E and Figure S5). On orthogonal projection (Figure 1E), QD emission was localized in broad spots, penetrating the cell interior. The maximum emission of the QD fluorescence signal was at 565 nm (Figure 1D), with about 5 nm red shift compared to measurement outside a cell (compare red line, Figure S4A). Such a shift may be explained by a small change near to the QD surface [17,18]. Here, it may simply indicate the slightly different ionization of Puf amino acids due to the pH of the particular cell compartment QD:Puf where they were located. A shift in QD emission maxima in response to pH is known from both in vitro and in vivo studies [19,20,21]. The autofluorescence of HeLa cells was checked in a control culture and was found to have a much lower intensity, with an emission maximum at about 530 nm (Figure 1E). The QD:Puf fluorescence lifetime (τ), determined by a FLIM experiment, was about 4 ns shorter than the 14 ns obtained before administration to cells (Figure S4B, Table S1). In both cases, a decay was fitted with two exponentials, which is a typical feature of QDs fluorescence [22]. Particular τ components and their respective relative amplitudes are provided in Supplementary Materials, Table S1. The individual τ may be attributed to electron transition from the conduction band and defects [23]. The shortening of τ again indicates some changes near to a QD surface [24].

The use of higher QD:Puf concentrations resulted in higher fluorescence accumulation inside cells, both for M and O fractions. When nanohybrid fractions containing extremely large aggregates (without a gel filtration pass) were used, no fluorescence corresponding to QDs was found inside cells (not shown). Administration of QD:Puf preparation, stored at −20 °C, resulted in significantly increased cell death, probably due to released Cd^2+^ toxicity. The preservation of QD:Puf nanohybrids was confirmed by fluorescence spectroscopy and gel electrophoresis. However, this verification cannot exclude the possibility that some parts of nanocrystals were destroyed in the process, even in the presence of glycerol as a cry-protectant. This highlights that a fresh preparation, or one stored at 4 °C, should be used in further studies.

The morphology of treated cells did not change, and their viability was further estimated quantitatively by MTT test (see paragraph 4). The dead cells contained a higher amount of QD:Puf, as evaluated from the total fluorescence intensity associated with a particular cell.

### 3.2. In Cell Localization of Nanohybrids

As the distribution of QD:Puf-related fluorescence was not equal across the cell body, we attempted more precisely to identify the actual cellular compartments accumulating the nanohybrids. In the cell, QDs may be accumulated by lysosomes [25], the endoplasmic reticulum (ER) [26] and the Golgi apparatus [27,28]. Some QDs were shown to enter mitochondria [28] and the nucleus [29]. QDs moving to the ER and lysosomes seems to be the default route—reaching the nucleus or mitochondria requires the presence of some specific ligands providing direction to these organelles [29]. QDs may be accumulated in a cell or removed by exocytosis. Some studies have shown that such discharge may be faster than endocytosis [30]. Similar cellular distribution was shown for other types of nanoparticles, such as carbon-based nanotubes or carbon quantum dots [31,32,33]. The classic way to identify target localization is to stain cells by compartment-specific fluorophores and to calculate co-localization with the signal of interest. Unfortunately, we found that, in the presence of stains, the QD signal diminished and could no longer be identified. This was caused by the relatively low quantum yield of QD:Puf in comparison to applied cell-compartment specific fluorophores and/or high local overload of the molecules required for efficient staining. The quantum yield of QD may be improved by the addition of an outer shell to the CdSe core; however, it also causes an increase in size and changes QD behavior—hence, this strategy was not applied.

Therefore, to identify QD:Puf target compartments, we stained the nucleus (with propidium iodide or DAPI) and membranes (plasma membrane by Laurdan stain as well as the ER and Golgi apparatus by specific immunostaining), and we compared the obtained pictures with separate images of QD fluorescence. QD:Puf emission was only clearly detected with immunostaining—the presence of other fluophores interfered with this measurement.

Stained cells (control and treated with QD:Puf) are shown in the  Supplementary Materials(Figures S6 and S7). QD-emission was localized in grain-like regions around the nucleus and further within the cell body (Figure 1E). The same shape and localization was observed for the Golgi apparatus and the ER (Figures S6 and S7); therefore, we suggest that the main localization of QD:Puf was in the broadly defined ER, including the Golgi network. The localization was confirmed by colocalization of QD:Puf emissions with specific immunostained regions (Figure S7E,F,O,P). The calculated Spearman correlation coefficients, quantifying colocalization, were 0.18 for QD:Puf and the Golgi apparatus, and 0.2 for QD:Puf and the ER. We also calculated the Manders’ colocalization coefficient, providing more insight into signal overlaps. The values were 0.403 for ER vs. QD:Puf, 0.191 for QD:Puf vs. ER, 0.117 for Golgi vs. QD:Puf, and 0.707 for QD:Puf vs. Golgi. These results suggest that a significant proportion of the QD:Puf was localized within the Golgi apparatus, and a smaller proportion within the ER. There was still some emission of QD:Puf that did not come from the ER or the Golgi body, which implies that a fraction of the nanoparticles was entrapped by endosomes. These endosomes may constitute part of the QD:Puf circulation in and out of the cell.

QD:Puf with a maximal diameter of about 15 nm (monomeric fraction) or 20–30 nm (aggregates fraction) were localized inside the ER cisternae (diameter of about 20–60 nm [34]). The accumulation of QD:Puf in ER suggests nanohybrid absorption by endocytosis mechanisms, which has been described for the uptake of other nanoparticles. This hypothesis is considered further in the next paragraph. The lack of efficient uptake of really large aggregates (more than 30 nm) suggests that phagocytosis was not involved in Puf:QD absorption.

### 3.3. Kinetics of Nanohybrid Absorption by Cells

**Figure 2:**
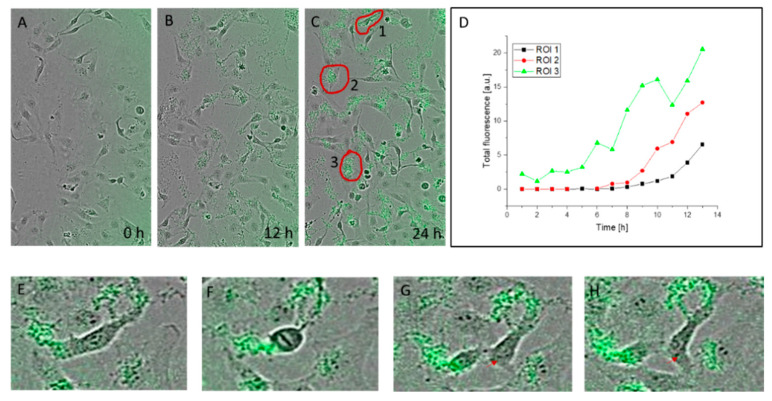
QD:Puf nanohybrid absorption by HeLa cells analyzed in real-time. The growth medium contained 10 nM Puf:QD. (**A**–**C**) are representative images of one spot, recorded before QD:Puf administration (**A**), after 12 h (**B**) and 24 h (**C**) of incubation with QD:Puf. Results of quantitative analysis of fluorescence changes in the region of interest (ROI) encircled in red in (**C**) are shown in (**D**). Note, that the green glimmer is a detection system artefact and was corrected during analysis. (**E**–**H**) show an example of a dividing cell with a clear indication of green fluorescence inheritance inside the cell interior (marked by a red arrow).

To learn more about the kinetics of the process, we analyzed QD:Puf nanohybrid absorption by HeLa cells using an automated live cell imaging system (IncuCyte). The cells were imaged every 2 h for 24 h of incubation. Several images were analyzed for QD:Puf accumulation as measured by cell-associated fluorescence increase. Examples of the data obtained are shown in Figure 2. In general, measurable accumulation of green fluorescence inside cells started after 4–6 h. This was much longer than the time necessary for endocytosis to occur, which is between seconds and minutes [35,36]. However, the observed fluorescence level was the result of uptake—with the limited sensitivity of the live cell imaging system, the first events of endocytotic uptake might have been missed. Therefore, what was observed in this experiment was the equilibrium state in which the uptake and removal of QD:Puf were no longer effective. This might have been due to uptake occurring faster than exocytosis (resulting in net accumulation), or to damage to the regulation mechanism, related to presence of QDs, resulting in a slowing of exocytosis. 

We investigated this issue further by comparing QD:Puf accumulation in the HeLa cells at 37 °C and at 22 °C. The lowering of temperature is a simple but efficient endocytosis inhibitor [37]. Interestingly, we found that, overall, cell-accumulated QD:Puf fluorescence was higher at the lower temperature (Figure S8A). It should be noted, however, that this emission was mostly localized at the plasma membrane (Figure S8B–D), not in the cell body. This suggests that endocytosis was actually the mechanism of QD:Puf absorption by the cell; when the plasma membrane fluidity was decreased at the lower temperature, the nanoparticles could not effectively enter the cell and accumulated on the cell surface.

The nanohybrids tended to aggregate in the medium, which was visible as clusters of grains. This may cause an impression of heterogeneity of distribution and lead to the conclusion that cells are differently susceptible to QD:Puf treatment. These clouds were mostly, but not only, associated with the cells. Their presence indicates that at least part of the “cell-associated” fluorescence measured by this method may be related to external fluorophores (QD:Puf not incorporated into the cell or only loosely associated with the plasma membrane). Part of the effect, however, may have resulted from slightly different focal planes of the cells. In CLSM, such clouds were not noted as the medium was exchanged before imaging. CLSM also did not confirm different sensitivity to QD:Puf among the cells.

For a few cases, we were able to track the inheritance of green fluorescence by dividing cells (Figure 2E–H). As the resolution of the method was too low to say if the inherited nanoparticles were inside the cell bodies or in the outer cloud, this was the only indication of the possibility of real inheritance. However, it is possible to state that QD:Puf did not disrupt crucial mechanisms responsible for proper cell functioning, such as cell proliferation.

### 3.4. Toxicity of Nanohybrids

**Figure 3:**
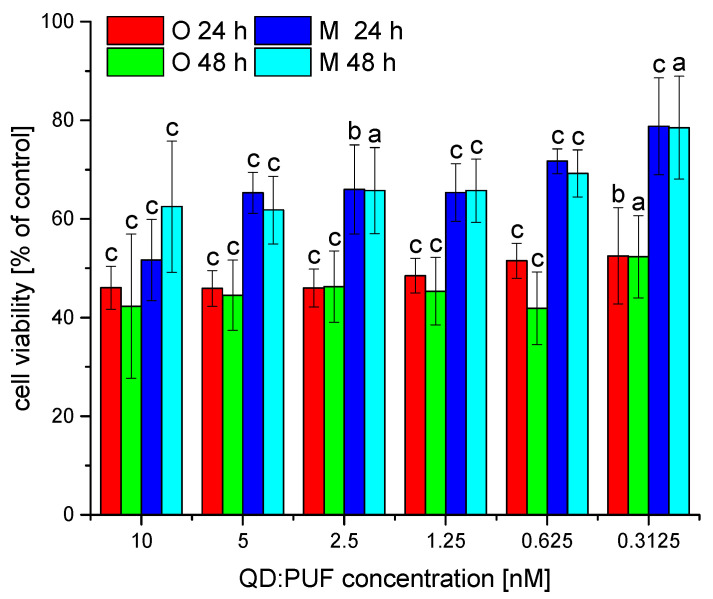
Changes in HeLa cell viability in response to decreasing concentration of QD:Puf in a growth medium. Two different fractions (O, oligomers, and M, monomers) were tested in a series of dilutions. Data show the ratio of average treatment to control, while error bars represent the standard deviation of % of control (calculated as the derivative of individual SD of treated and control samples). Letters a, b and c indicate a statistically significant difference between given conditions and control (no QD:Puf) at *p* < 0.05, *p* < 0.01 and *p* < 0.001, respectively.

Visual inspection of cell cultures suggested that, in the presence of nanohybrids. the number of dead cells increased. Therefore, we performed quantitative toxicity tests. The results indicated a decrease in viability of about 20–60% of the control (Figure 3), depending on the incubation time, concentration and QD:Puf fraction used. The toxicity of larger nanohybrids (fraction O) was significantly higher compared to the monomeric QD:Puf preparation (fraction M). A lower concentration of QD:Puf increased viability. This observation was more pronounced for the M fraction of nanohybrids. Prolonged incubation (24 h vs. 48 h) further decreased viability, especially for the O fraction; however, this change was not statistically significant. The changes in cell viability corresponded to the cell multiplication factor, calculated for selected QD:Puf concentrations (see Figure S9). The cell number almost duplicated over 24 h in the control conditions, while, for 10 nM QD:Puf, this parameter was reduced by about 25%. Zhang and coworkers [38] also compared the toxicity of Qds. In this study, HepG2 (human hepatoma) cells were exposed to CdTe Qds which varied by diameter [38]. Smaller nanoparticles were more toxic, an opposite finding to our results. This might be explained, however, by the different range of sizes tested, suggesting that there might be an optimal size for compatibility with cellular studies.

QDs, especially QDs composed of cadmium, are well-known to induce cytotoxicity. The mechanism is connected to the direct toxicity of Cd^2+^ ions released during QD decomposition. Such a process is more likely when the QD surface is directly accessible to the cytoplasm and other cell fluids, especially at low pH in lysosomes. Moreover, the free cadmium ions may originate from the synthesis of QDs if not properly purified [38]. Here, any free Cd ions would be separated in the gel filtration step. The additional outer shell, which here was a Puf protein, may provide protection against QD degradation. The protein will form a barrier between the nanocrystal surface and the potentially reactive cellular interior (e.g., lysosomes of low pH). We cannot, however, completely exclude the mechanism of toxicity related to decomposition of QDs, primarily due to changes noted in QD fluorescence, indicating some influence of the exterior on the QD surface. Nevertheless, the decomposition would be increased with time, which should also greatly increase toxicity at 48 h over 24 h. Since we did not observe this, it is reasonable to assume that this may not be the main toxicity mechanism here. It is also possible that QDs induce the production of reactive oxygen species (ROS) [39] or disturb natural processes by changing the ER interior specificity due to over-accumulation. For ROS production, the cell may respond with induction of antioxidant pathways [40,41], which acts against increased toxicity. Over-accumulation may also reflect the balance between uptake and removal. After 24 h, there might be too low a QD:Puf concentration in the medium to significantly increase the QD:Puf concentration in the ER. We tested this hypothesis by repeating the toxicity tests at a lowered starting confluence (1 × 10^3^ cells per well). Counterintuitively, the resulting toxicity was slightly lower (Figure S10). This indicates that, at least partially, the metabolites of QD might be responsible for the observed toxicity; as the cell number decreases, the concentration of metabolites also decreases, and so do the toxic effects.

### 3.5. Other Cells

**Figure 4:**
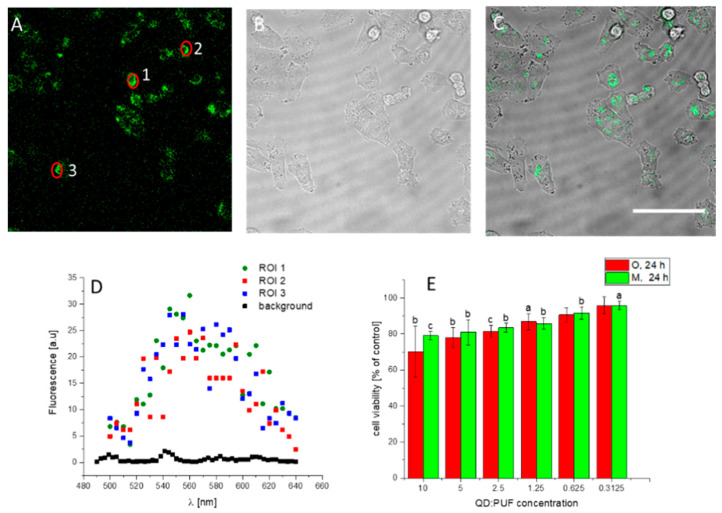
Puf:QD nanohybrids visualized inside A375 cells after 24 h of incubation. The growth medium contained 10 nM of QD:Puf. CLSM images (**A**) fluorescence emission (excitation 450 nm, emission 550–650 nm), (**B**) bright-field and (**C**) image overlay. The bar is 65 μm. (**D**) emission spectra recorded for ROIs marked as the red circle in the image (**A**), background and control emission detected in untreated, control cells. (**E**) Changes in HeLa cell viability in response to decreasing concentration of Puf:QD in a growth medium. Two different fractions (O, oligomers, and M, monomers) were tested in a series of dilutions. Data show the ratio of average treatment to control, while error bars represent the standard deviation of % of control (calculated as the derivative of individual SD of treated and control samples). Letters a, b and c indicate a statistically significant difference between given conditions and control (no QD:Puf) at *p* < 0.05, *p* < 0.01 and *p* < 0.001, respectively.

The HeLa cell line is a model cell line used widely for research purposes and is often thought of as a first choice option. However, it might be argued that the QD:Puf uptake was due to its specific properties, including a high mutation rate or plasma membranes with a broad set of receptors [42]. Therefore, we checked the QD:Puf performance in the A375 melanoma cell line (Figure 4), to get an idea of the possible performance of the nanohybrids in other cells. This line was selected due to its different morphology and metabolism in comparison to Hela cells. QD:Puf preparation was also absorbed from the culture medium and accumulated inside these cells. Fluorescence emission maxima were the same as in the HeLa cells (Figure 4D). The toxicity (Figure 4E) was lower than observed in the HeLa cells, which might be connected to the different metabolism of these types of cells. The metabolism differences were notable in terms of the doubling rate, which was faster for the A375 cells [43] in comparison to the HeLa cells. This might lead to faster QD:Puf accumulation. Melanoma cells are also known to accumulate cholesterol due to disrupted cholesterol homeostasis [44] which results in changes in membrane composition. In general, the cell membranes of cancer cells are changed differently in different cancer types. These observations suggest that both the metabolism rate and membrane composition are important for nanoparticle uptake and their toxicity.

## 4. Conclusions

QD:Puf preparation was shown to be stable during the experiments and the particles were successfully delivered inside HeLa and A375 melanoma cells by simple uptake from the growth medium. The accumulation of measurable fluorescence intensity required at least 6–10 h exposure, suggesting that the net observed signal was the result of endo- and exocytosis. Both monomeric QD:Puf and the relatively large oligomers of QD:Puf may be accumulated inside cells; however, the presence of oligomers resulted in higher cytotoxicity. QD fluorescence localized in the ER, with regions of higher intensity (probably the Golgi apparatus). Therefore, it is possible that the observed toxicity was not (or not only) due to Cd^2+^ ion release, but due to disturbance of ER function.

The fluorescence emission maximum was red-shifted inside the cells and the fluorescence lifetime was shortened, indicating some influence of the cell microenvironment on the QD surface. These observations might be an indirect effect, related to changes in the covering protein, Puf, and to amino acid ionization.

In sum, we have demonstrated that QD cover, composed of long peptides, assures good nanoparticle performance in cell studies. This makes Puf and its derivatives the next option in fluorescent probes and sensor development. In such applications, a Puf-based (or other peptide-based) cover offers places for secondary chemistry to occur on the nanoparticle surface. A protein-based cover may provide protection of the nanoparticle surface from the reactive cellular interior, resulting in resistance to decomposition and release of toxic cadmium ions. In addition, a peptide might be modified by the addition of special tags using protein engineering before nanohybrid assembly.

## Data Availability

All data are available in the manuscript and Supplementary Materials. Crude data presented in this study are available on request from the corresponding author.

## Supplementary Materials

The following supporting information can be downloaded at: www.mdpi.com/article/10.3390/nano12183174/s1. Figure S1: Representative TEM micrograph of as-synthesized raw CdSe QDs; Figure S2: A representative chromatogram of Puf:QD separation on Superdex 200 10/300 column. The elution was analyzed by absorption at 280 nm (QD and protein absorption) and 550 nm (QD exclusive absorption). Fractions selected for further tests (O, oligomers and M, monomers) are marked. The last peak (at about 17.5 mL) is an excess of Puf protein, not assembled with QD; Figure S3: Changes in the QD:Puf emission during 24 h incubation in a cell growing medium. Temperature of incubation was 37 °C. Background emission of the medium is subtracted; Figure S4: Absorption spectrum (A) and fluorescence decay (B) recorded for QDs in chloroform, QD:Puf preparation before admission to cells and for QD:Puf inside a cell (excitation 471 nm). Fluorescence decays for QDs in chloroform and QD:Puf preparation recorded in a cuvette, at fluorescence emission maximum. The decay in a cell recorded using FLIM-CLSM setup and fluorescence range 546–611 nm; Figure S5: Consecutive original images (overlay of bright field and QD emission, as well as corresponding images with QD emission only) of Z-stack, presented in Figure 1E of the main manuscript. Z spacing was 300 nm. Scale bar is 15 μm. Images were not subjected to deconvolution or any other modification, except contrast adjustment; Figure S6: Hela cells (control, A–C, and incubated with QD:Puf, D–G) stained with propidium iodide (red, A, C, F, G) and Laurdan (green, B–E). Image C is the overlay of A, B and bright field for control cells. Images G is overlay of F and bright field for QD:Puf treated cells; Figure S7: Hela cells: control (A–D, I–L) and QD:Puf treated (E–F, M–P), immnostained for visualization of Golgi apparatus (A–H) and endoplasmic reticulum (G–M). QD:Puf emitted in green (F, N), secondary antibodies, labelled with Alexa 647, gave signal in red channel (A, E) while nucleuses of the cells were stained with DAPI and emitting in blue range (B,F). For QD:Puf treated cells Dapi staining was omitted, as in iterferred with QD:Puf detection. Images D and E are the overlay of CLSM images and respective and bright field (C, G); Figure S8: Changes in QD:Puf (10 nM) uptake by HeLa cells as a result of lower incubation temperature. (A) Comparison of total cell accumulated QD:Puf fluorescence (points - individual cell values, bars - average for whole measurement, error bars—standard error) and (B–D) representative images (QD:Puff emission, brigh field and overlay, representatively) showing particles adsorption mainly at cell surface; Figure S9: Proliferation index, calculated for control and QD:Puf treated cells, expressed as cell number multiplication after 24 h. Data show average ± SE; Figure S10: Comparison of changes in HeLa cells viability in response to decreasing concentration of QD:Puf in a growth medium for two starting cell confluence values, 5 × 10^3^ cells/well (control confluence) and 1 × 10^3^ cells/well (lowered confluence). Data show ratio of averages treatment to control, while error bars represent the standard deviation of % of control (calculated as derivative of individual SD of treated and control samples). Stars indicate a statistically significant difference between viability at control and lowered confluence; * *p* < 0.05 and *** *p* < 0.001; Table S1. Individual τ components and their relative amplitude (A), fitted for fluorescence decay curves shown in Figure S3.
