# Supplementary material for: QD:Puf Nanohybrids Are Compatible with Studies in Cells
Source: Nanomaterials (Basel). 2022 Sep 13;12(18):3174. doi: 10.3390/nano12183174 (PMC9506232; doi:10.3390/nano12183174)
Supplement: Supplementary file 1 [file nanomaterials-12-03174-s001.zip › nanomaterials-1837434-supplementary.pdf]

nanomaterials

Supplementary Materials

QD:Puf Nanohybrids Are Compatible with Studies in Cells

Karolina Wójtowicz [1], Magda A. Antoniak [2], Martyna Trojnar [3], Marcin Nyk [2], Tomasz Trombik [1,4] and Joanna Grzyb [3,*]

1 Department of Biotransformation, Faculty of Biotechnology, University of Wrocław, ul. F. Joliot-Curie 14a, 50-383 Wrocław, Poland
2 Advanced Materials Engineering and Modelling Group, Faculty of Chemistry, Wrocław University of Science and Technology, Wybrzeże Wyspiańskiego 27, 50-370 Wrocław, Poland
3 Department of Biophysics, Faculty of Biotechnology, University of Wrocław, ul. F. Joliot-Curie 14a, 50-383 Wrocław, Poland
4 The Chair and Department of Biochemistry and Molecular Biology, Medical University of Lublin, ul. Chodzki 1, 20-093 Lublin, Poland
* Correspondence: joanna.grzyb@uwr.edu.pl

Table S1. Individual τ components and their relative amplitude (A), fitted for fluorescence decay curves shown in Figure S3.

	τ1 [ns]	A1	τ2 [ns]	A2	τ3 [ns]	A3	τI [ns]	τA [ns]
QD in chloroform	3.9	0.6	17.5	0.35	65	0.05	11.8	27.7
QD:Puf	2.5	0.72	18.6	0.28	-	-	7.0	14.5
QD:Puf inside a cell	2.0	0.57	7.5	0.42	-	-	2.9	4.3

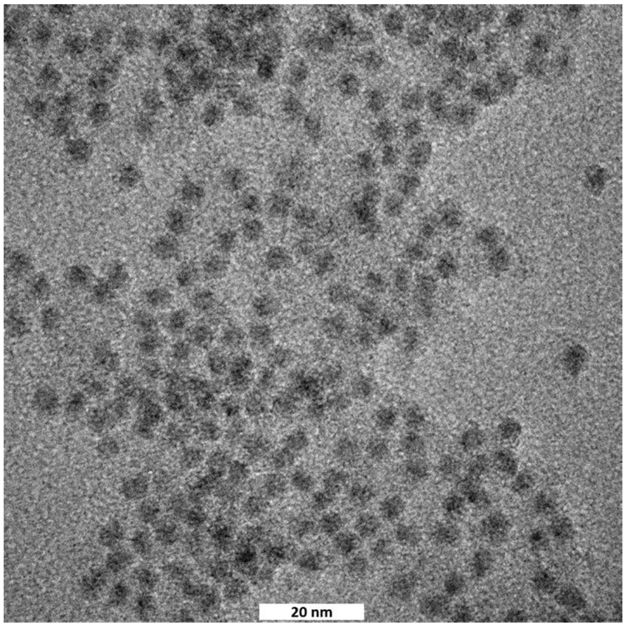

Figure S1. Representative TEM micrograph of as-synthesized raw CdSe QDs.

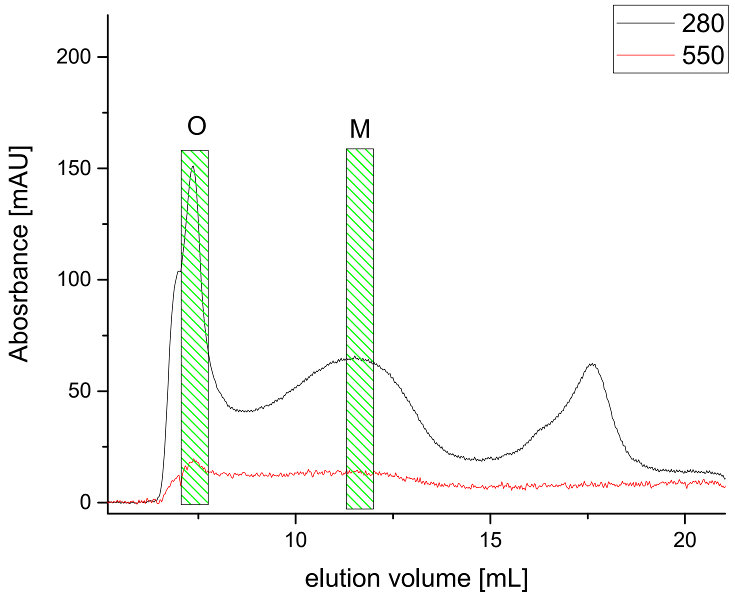

Figure S2. A representative chromatogram of Puf:QD separation on Superdex 200 10/300 column. The elution was analyzed by absorption at 280 nm (QD and protein absorption) and 550 nm (QD exclusive absorption). Fractions selected for further tests (O, oligomers and M, monomers) are marked. The last peak (at about 17.5 mL) is an excess of Puf protein, not assembled with QD.

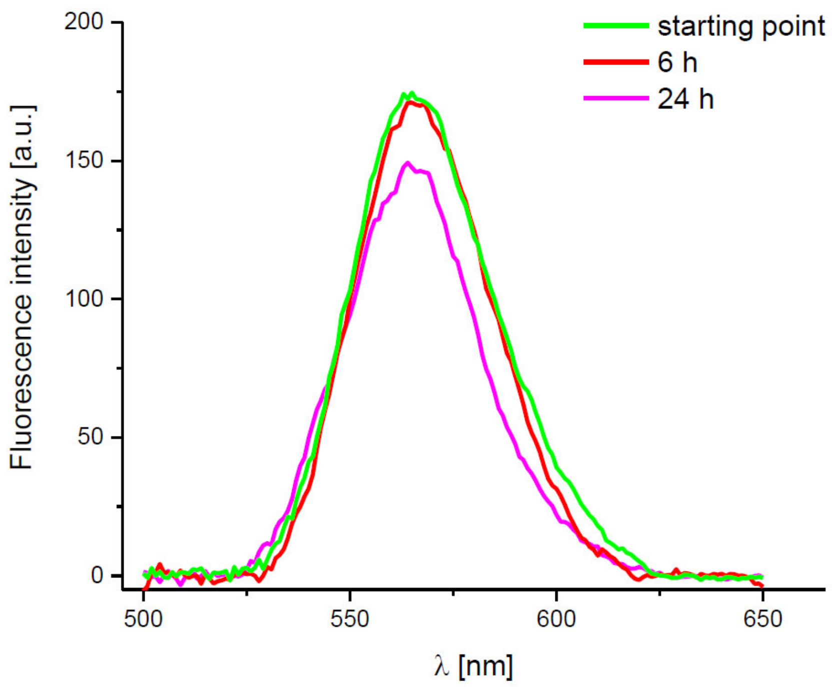

Figure S3. Changes in the QD:Puf emission during 24 h incubation in a cell growing medium. Temperature of incubation was 37 °C. Background emission of the medium is subtracted.

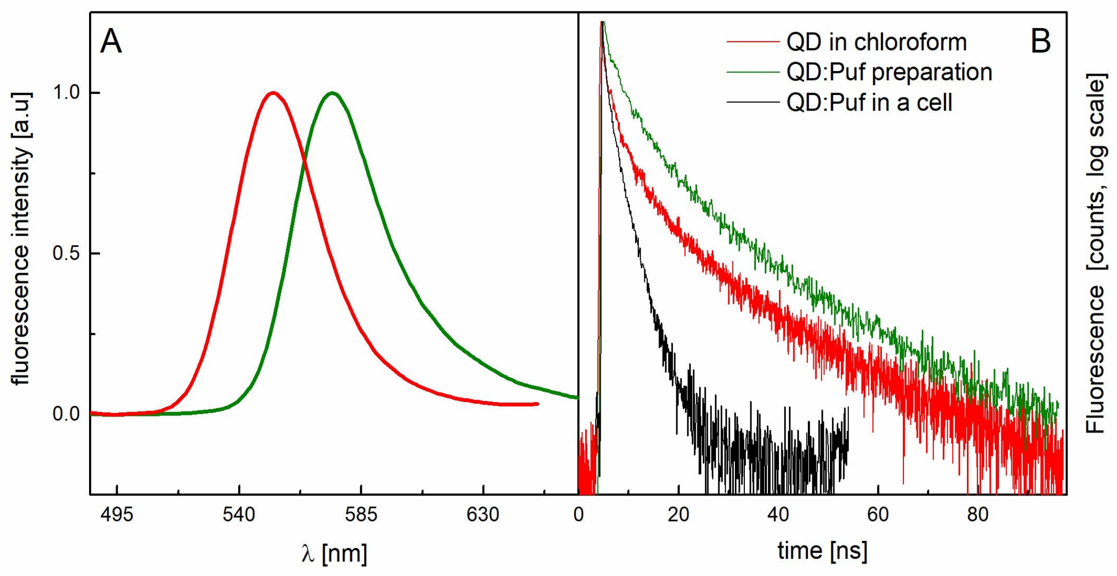

Figure S4. Absorption spectrum (**A**) and fluorescence decay (**B**) recorded for QDs in chloroform, QD:Puf preparation before admission to cells and for QD:Puf inside a cell (excitation 471 nm). Fluorescence decays for QDs in chloroform and QD:Puf preparation recorded in a cuvette, at fluorescence emission maximum. The decay in a cell recorded using FLIM-CLSM setup and fluorescence range 546–611 nm.

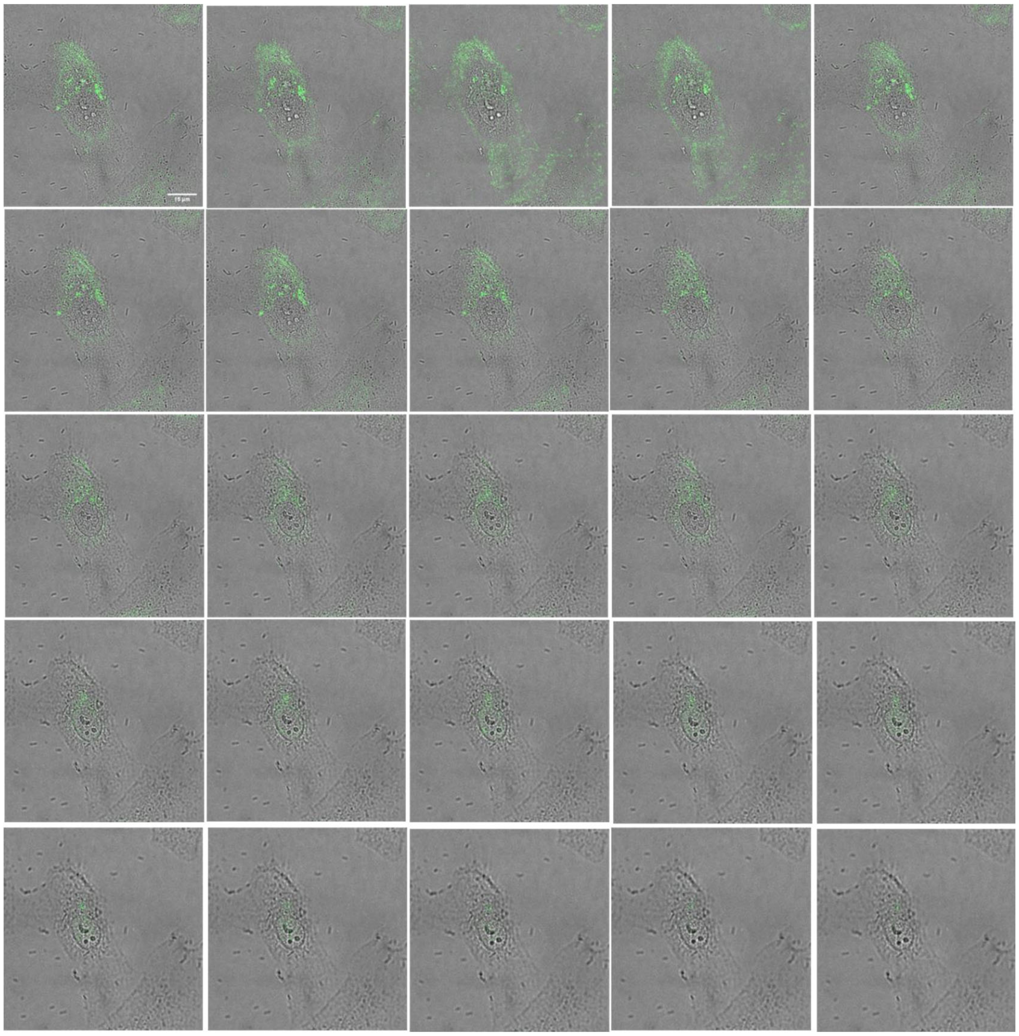

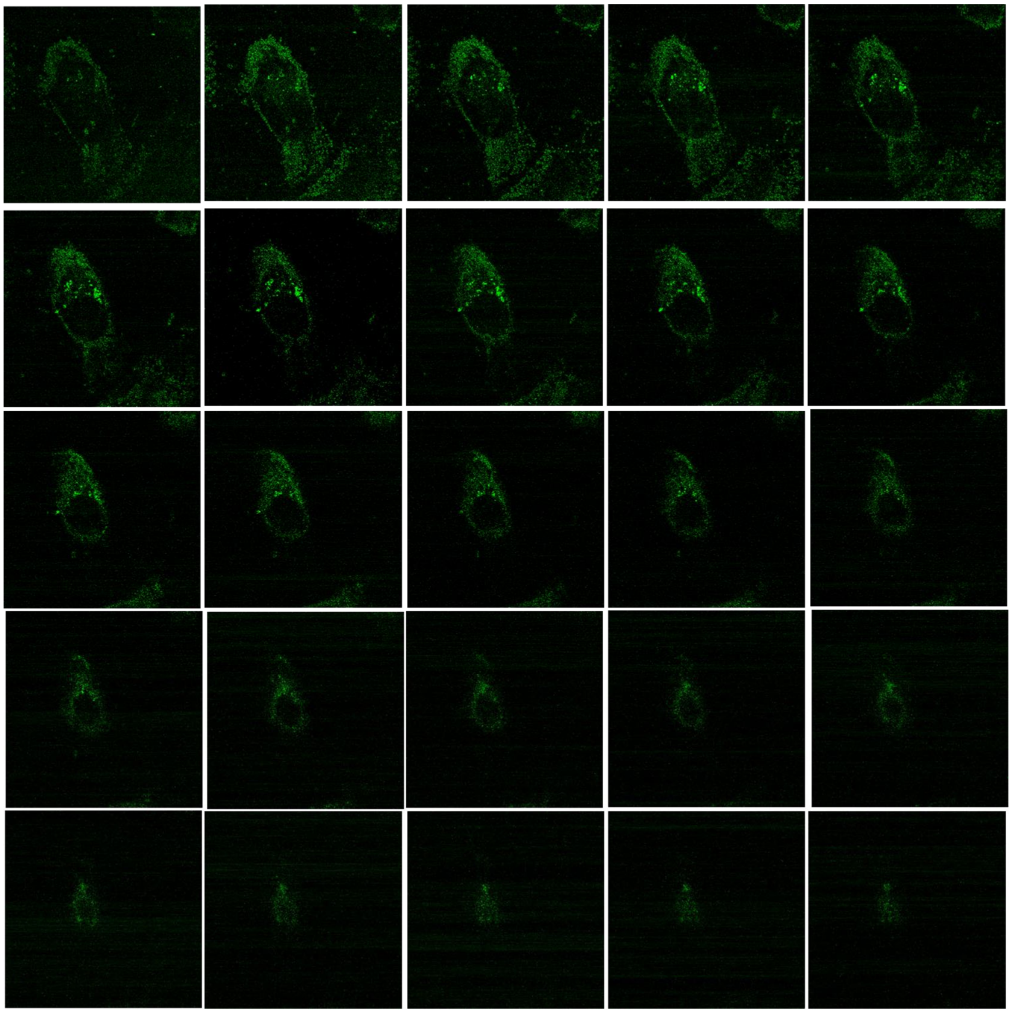

Figure S5. Consecutive original images (overlay of bright field and QD emission, as well as corresponding images with QD emission only) of Z-stack, presented in Fig. 1E of the main manuscript. Z spacing was 300 nm. Scale bar is 15 µm. Images were not subjected to deconvolution or any other modification, except contrast adjustment.

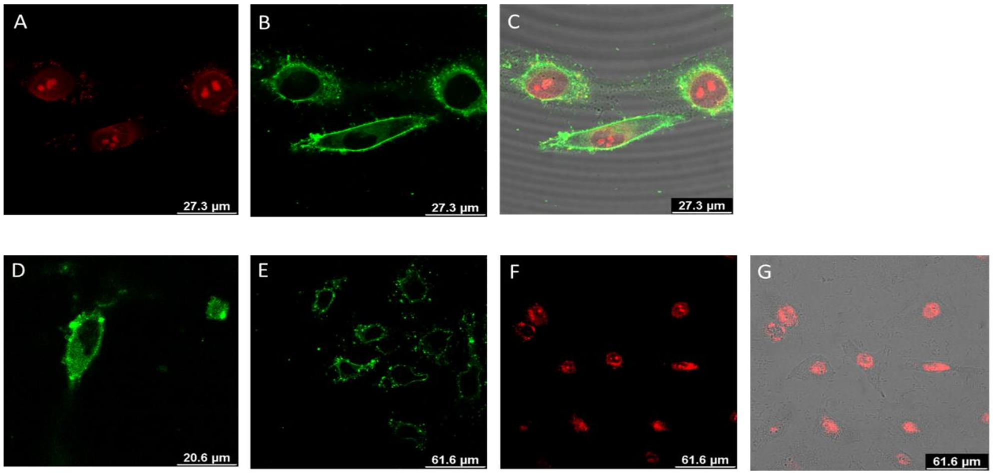

Figure S6. Hela cells (control, **A**–**C**, and incubated with QD:Puf, **D**–**G**) stained with propidium iodide (red, **A**, **C**, **F**, **G**) and Laurdan (green. **B**–**E**). Image **C** is the overlay of **A**, **B** and bright field for control cells. Images **G** is overlay of **F** and bright field for QD:Puf treated cells.

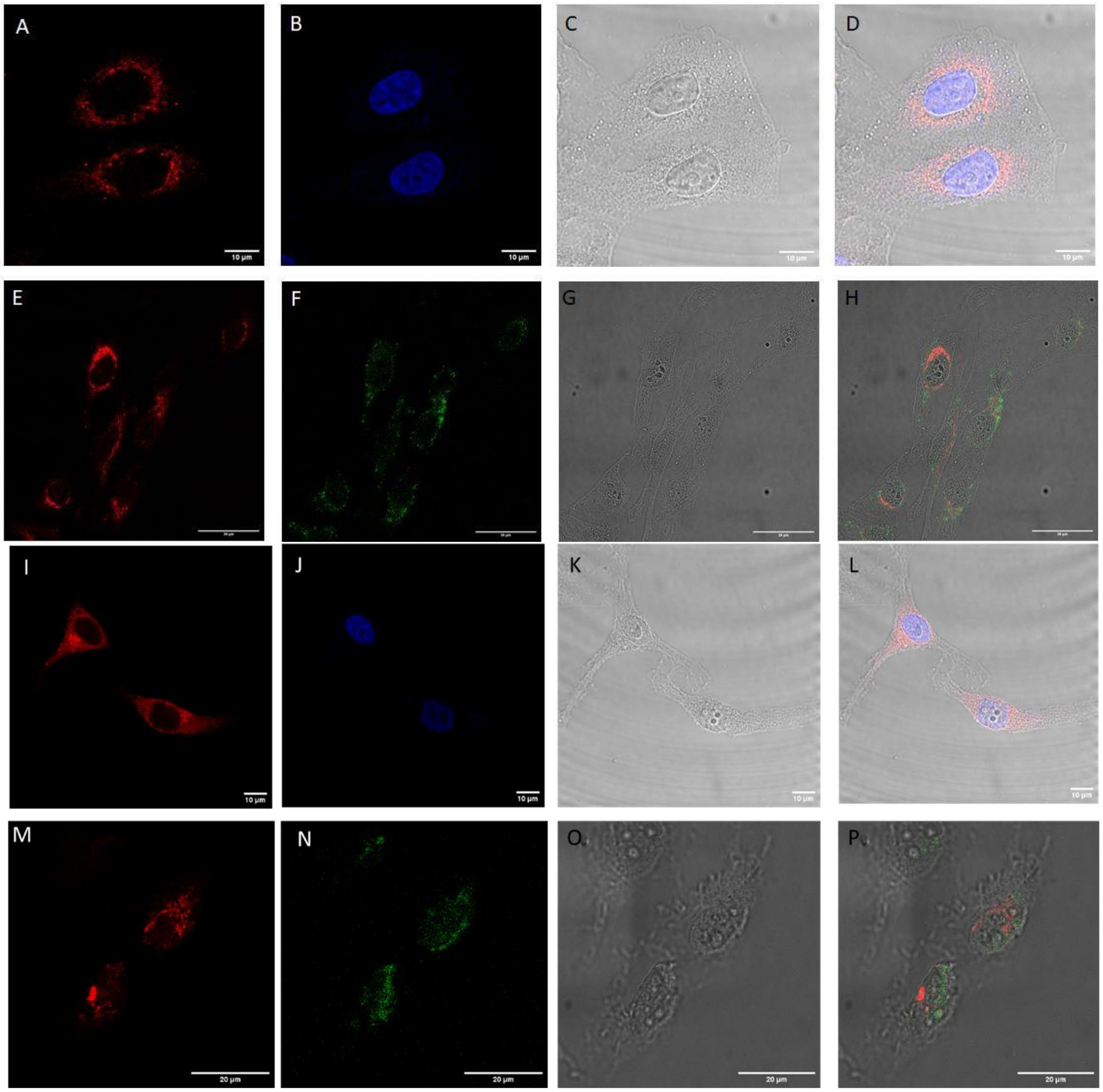

Figure S7. Hela cells: control (**A**–**D**, **I**–**L**) and QD:Puf treated (**E**–**F**, **M**–**P**), immnostained for visualization of Golgi apparatus (**A**–**H**) and endoplasmic reticulum (**G**–**M**). QD:Puf emitted in green (**F**,**N**), secondary antibodies, labelled with Alexa 647, gave signal in red channel (**A**, **E**) while nucleuses of the cells were stained with DAPI and emitting in blue range (**B**, **F**). For QD:Puf treated cells Dapi staining was omitted, as in iterferred with QD:Puf detection. Images **D** and **E** are the overlay of CLSM images and respective and bright field (**C**, **G**).

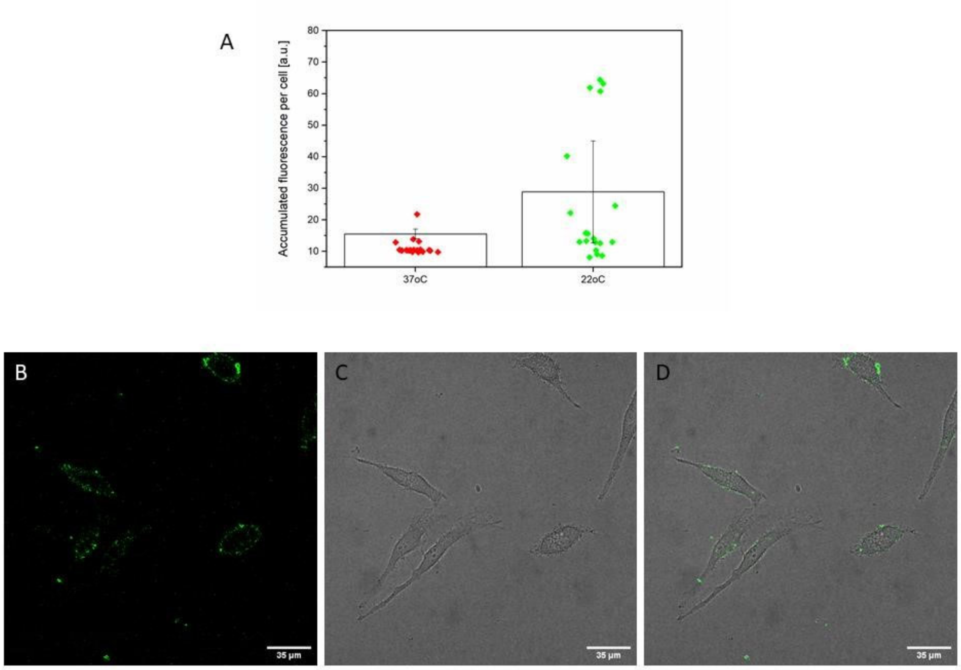

Figure S8. Changes in QD:Puf (10 nM) uptake by HeLa cells as a result of lower incubation temperature. (**A**) Comparison of total cell accumulated QD:Puf fluorescence (points - individual cell values, bars - average for whole measurement, error bars - standard error) and (**B**–**D**) representative images (QD:Puff emission, brigh field and overlay, representatively) showing particles adsorption mainly at cell surface.

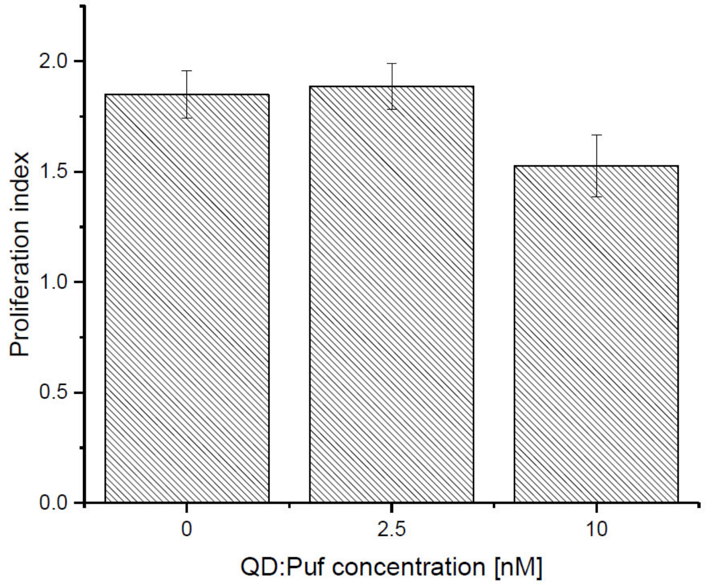

Figure S9. Proliferation index, calculated for control and QD:Puf treated cells, expressed as cell number multiplication after 24 h. Data show average ± SE.

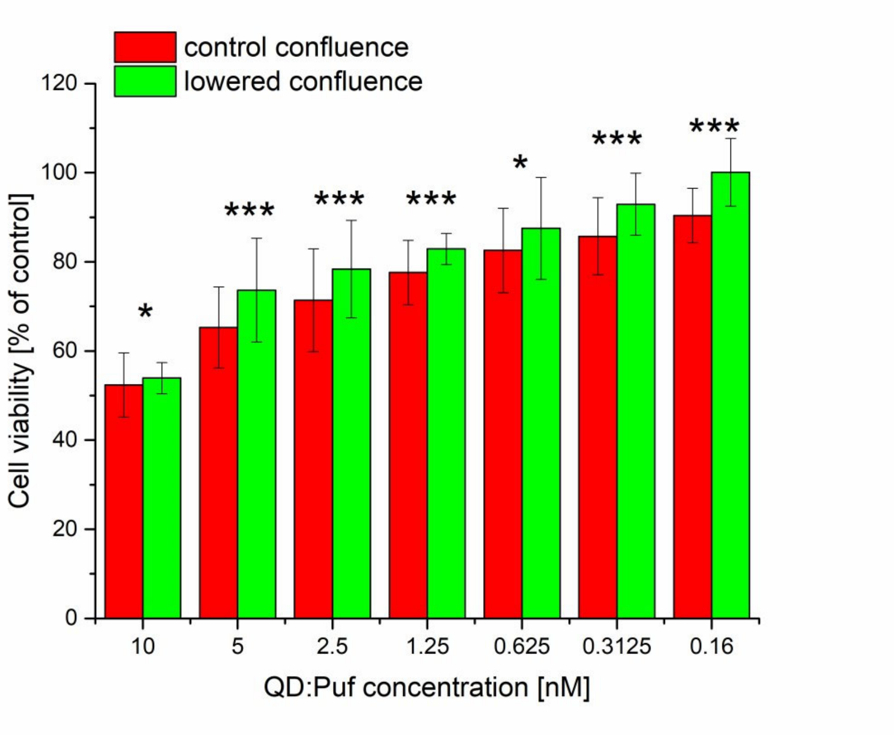

Figure S10. Comparison of changes in HeLa cells viability in response to decreasing concentration of QD:Puf in a growth medium for two starting cell confluence values, 5×10^3 cells/well (control confluence) and 1×10^3 cells/well (lowered confluence). Data show ratio of averages treatment to control, while error bars represent the standard deviation of % of control (calculated as derivative of individual SD of treated and control samples). Stars indicate a statistically significant difference between viability at control and lowered confluence; * $p < 0.05$ and *** $p < 0.001$.
